# Molecular and cellular mechanisms underlying brain metastasis of breast cancer

**DOI:** 10.1007/s10555-020-09881-y

**Published:** 2020-05-13

**Authors:** Mari Hosonaga, Hideyuki Saya, Yoshimi Arima

**Affiliations:** 1 Division of Gene Regulation, Institute for Advanced Medical Research, Keio University School of Medicine, 35 Shinano-machi, Shinjuku-ku, Tokyo, 160-8582 Japan; 2 Breast Medical Oncology Department, Cancer Institute Hospital of Japanese Foundation for Cancer Research, 3-8-31 Ariake, Koto-ku, Tokyo, 135-8550 Japan

**Keywords:** Breast cancer, Brain metastasis, Astrocyte, STAT3, PI3K-Akt, xCT

## Abstract

Metastasis of cancer cells to the brain occurs frequently in patients with certain subtypes of breast cancer. In particular, patients with HER2-positive or triple-negative breast cancer are at high risk for the development of brain metastases. Despite recent advances in the treatment of primary breast tumors, the prognosis of breast cancer patients with brain metastases remains poor. A better understanding of the molecular and cellular mechanisms underlying brain metastasis might be expected to lead to improvements in the overall survival rate for these patients. Recent studies have revealed complex interactions between metastatic cancer cells and their microenvironment in the brain. Such interactions result in the activation of various signaling pathways related to metastasis in both cancer cells and cells of the microenvironment including astrocytes and microglia. In this review, we focus on such interactions and on their role both in the metastatic process and as potential targets for therapeutic intervention.

## Introduction

Cancer metastasis to the brain occurs predominantly in cases of lung adenocarcinoma, breast carcinoma, and melanoma [1]. Brain metastases thus develop in 10% to 30% of women with metastatic breast cancer [2]. Local therapeutic approaches such as surgery and radiotherapy have proved effective for metastatic brain tumors, and systemic therapies that control extracranial disease are improving. However, specific therapies that target brain metastases in breast cancer patients have not been established, and the prognosis of such patients therefore remains poor. Identification of the cellular and molecular mechanisms underlying brain metastasis of breast cancer is likely to provide a basis for the prevention or treatment of such disease. In this review, we focus on the signaling processes related to brain metastasis of breast cancer and discuss the prospects for and clinical implications of targeting the molecules involved.

## Brain metastasis

Metastatic brain tumors develop when cancer cells from a primary tumor elsewhere in the body reach the brain via the bloodstream and begin to proliferate. Metastases have been detected in the frontal, temporal, parietal, and occipital lobes, the cerebellum, and other locations in the brain. Brain metastases are diagnosed by contrast-enhanced magnetic resonance imaging or computed tomography. The neurological symptoms of brain metastases are largely the result of compression of the brain tissue at the site of tumor formation and of increased intracranial pressure, and they include headache, nausea, and vomiting, epileptic seizures, dizziness, limb paralysis, convulsions, impaired vision, and speech problems. There are three therapeutic options for metastatic brain tumors: radiation therapy, surgery, and treatment with anticancer drugs including molecularly targeted agents. Serious neurological symptoms often disturb the treatment of tumors with anticancer drugs. On the basis of the number of metastases and the predicted prognosis, patients are treated so as to control tumor growth, to ameliorate neurological symptoms, or to improve quality of life.

## Biology of brain metastasis of breast cancer

As mentioned above, brain metastases commonly arise in patients with lung cancer, breast cancer, or melanoma and are associated with a poor survival outcome [3]. In addition to the brain, breast cancer cells metastasize to bone, liver, lung, and distant lymph nodes [4], with bone being the most common metastatic site for breast cancer. Breast cancer is divided into various subtypes on the basis of the expression status of human epidermal growth factor receptor 2 (HER2) and of estrogen (ER) and progesterone (PR) receptors by immunohistochemical staining or gene expression profiles [5, 6]. These breast cancer subtypes have been found to possess different gene signatures, to rely on different signaling pathways for metastasis, and to show different metastatic site preferences [7]. Patients with HER2-positive breast cancer or triple-negative (HER2^−^ ER^−^ PR^−^) breast cancer (TNBC) have a higher risk of brain metastasis compared with those with the luminal subtype (ER^+^ or PR^+^) of breast cancer. The frequency of brain metastasis is thus as high as 20% to 30% in HER2-positive breast cancer and TNBC but is < 10% in luminal breast cancer [4].

Despite recent advances in the treatment of primary breast tumors, the prognosis for breast cancer patients with brain metastases remains poor overall—although the prognosis of such patients is actually heterogeneous, with some individuals achieving a better survival outcome than others [8]. Several prognostic scores have been developed to estimate the survival of breast cancer patients with brain metastases, with the graded prognostic assessment (GPA) being a relatively new prognostic index for patients with brain metastases [8–10]. These prognostic scores are based on the clinical and histopathologic factors, including performance status, age at diagnosis of brain metastasis, breast cancer subtype, number of brain metastatic lesions, and the presence and status of extracranial disease. Breast-GPA and modified breast-GPA have been found to accurately predict overall survival for breast cancer patients with brain metastases (*p* < 0.001 for both scores) [10], and modified breast-GPA has been generally used in clinic as a prognostic scoring tool.

## Microenvironment of metastatic brain tumors

Cancer metastasis is a multistep process that includes local invasion of a primary tumor into the surrounding tissue, intravasation of tumor cells and their dissemination in the circulation, extravasation of the circulating cancer cells at distant sites, and the colonization by these cells of such sites, giving rise to the formation of micro- and then macroscopic metastases [11, 12]. Organ-specific colonization is dependent on the interaction between cancer cells and their microenvironment. Colonization of the brain by cancer cells is thus coordinated by molecular pathways involving the cancer cells as well as surrounding stromal cells, immune cells, and extracellular matrix [13], all of which contribute to the tumor microenvironment and regulate the biology of tumors in the brain [14].

The percentage of intratumoral lymphocytes has been found to be an independent predictor of a pathological complete response to neoadjuvant chemotherapy for breast cancer [15]. Tumor-infiltrating lymphocytes (TILs) also appear to be a key factor in the development of brain metastases [16], even though lymphocytes are rarely found in the brain parenchyma. A retrospective study found that TILs were present in > 90% of brain metastases of patients with breast cancer [17]. Although the immune cell types present in the brain differ from those in other organs, it appears that the brain is not as “immune privileged” as was once thought, raising the possibility that primary brain tumors and brain metastases might be successfully targeted by immunotherapy [18].

Astrocytes are abundant cells in the brain and play a role in tissue homeostasis, including maintenance of the blood-brain barrier. Circulating cancer cells need to pass through this barrier (extravasation) before they can colonize and proliferate in the brain. Although most extravasated cancer cells die, the surviving cells bind to the external surface of brain capillaries and grow as a sheath around the vessels [19]. Astrocytes have long been recognized as a key stromal component of both primary and metastatic brain tumors, and they have been found to have both tumor-killing and tumor-promoting effects, likely reflecting the fact that these cells exist as distinct subtypes with distinct functions [20]. For example, astrocytes produce plasminogen that induces apoptosis of cancer cells, whereas astrocyte-derived cyclic GMP-AMP synthase (cGAS) and microRNAs (miRNAs) delivered to tumor cells via gap junctions or exosomes have been shown to promote the formation of brain metastases [21]. Several astrocyte subtypes have been identified in both mice [22] and humans [23]. Neuroinflammation and ischemia are associated with the generation of two different populations of reactive astrocytes termed A1 and A2, with A1 astrocytes being regarded as proinflammatory and A2 astrocytes being thought to promote tissue repair through the production of neurotrophic factors [24, 25]. It is thought that most tumor-associated astrocytes are likely to be of the A2 subtype [26]. It remains unclear whether A1 and A2 astrocytes are able to undergo interconversion, and it is possible that other phenotypes also exist.

Both primary and metastatic brain tumors are influenced by the distinct biology of the brain microenvironment characterized by its unique cell types, anatomic structures, metabolic constraints, and immune properties. Given the important role of the tumor microenvironment in both the metastatic process and response to treatment, characterization of the relation between tumor cells and their microenvironment in the brain is likely to inform the development of new approaches to the prevention or therapy of primary brain tumors and brain metastases. We will describe several signal transduction molecules and their regulatory mechanisms that are associated with both tumor cells and brain microenvironmental cells.

## Molecular signals in the regulation of brain metastasis

### STAT3 signaling pathway

**Fig. 1:**
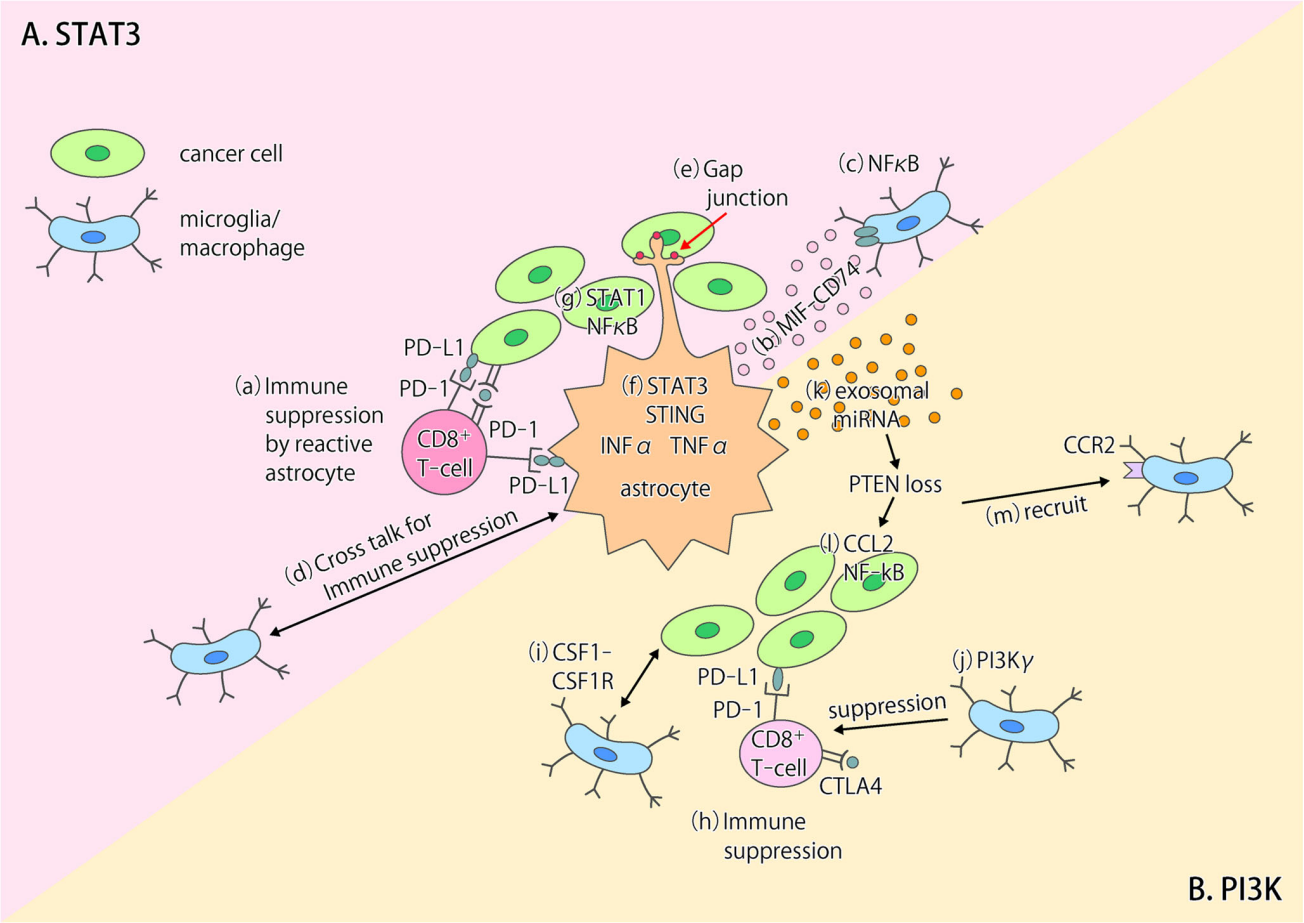
Models for the interactions between tumor cells and the tumor microenvironment in the brain. **a** The STAT3 and NF-κB signaling pathways play key roles in tumor-associated astrocytes in brain metastases. (**a**) Reactive astrocytes show phosphorylation of STAT3 and expression of PD-L1, which may contribute to the suppression of CD8^+^ T cell function [26]. (**b**) Reactive astrocytes positive for STAT3 activation increase the number of CD74^+^ microglia-macrophages in brain metastases through production of the CD74 ligand MIF and consequent activation of the MIF-CD74 axis [26]. (**c**) The NF-κB pathway is activated in CD74^+^ microglia-macrophages [26]. (**d**) Cross talk between microglia-macrophages and astrocytes contributes to establishment of an immunosuppressive environment in primary brain tumors [28]. (**e**) Cancer cells transfer cGAMP to astrocytes through Cx43-PCDH7 gap junctions, resulting in activation of the cGAS-STING pathway in the latter cells [32]. (**f**, **g**) Production of IFN-α and TNF-α by astrocytes induces activation of STAT1 and NF-κB pathways in cancer cells and thereby supports brain metastasis [32]. **b**. The PI3K-Akt signaling pathway plays a key role in brain metastases of breast cancer cells. (**h**) PI3K activation up-regulates PD-L1 and CTLA4 expression in cancer cells [33]. (**i**) Cross talk between cancer cells and macrophages results in activation of PI3K and CSF1-CSF1R signaling in macrophages [33]. (**j**) PI3Kγ signaling in macrophages inhibits NF-κB activation and promotes immune suppression in head and neck cancer [38]. (**k**) Loss of PTEN expression in cancer cells is induced epigenetically by exosomal miRNAs released from astrocytes [41]. (**l**, **m**) PTEN loss results in increased expression of the chemokine CCL2 and activation of NF-κB signaling in cancer cells as well as in the consequent CCR2-dependent recruitment of macrophages [41]

The Janus kinase (JAK)–signal transducer and activator of transcription (STAT) pathway is a key signaling mechanism activated by the interaction of cytokines and growth factors with their receptors. The induction of A2 astrocytes by ischemia is associated with scar formation [25], and the STAT3 signaling pathway plays an important role in astrocytic scar formation, which promotes axon regeneration [27]. Most astrocytes found in brain metastases were recently shown to express the phosphorylated (activated) form of STAT3 (pSTAT3) [26], suggesting that STAT3 signaling also plays a key role in the tumor-associated cells (Fig. 1a). Indeed, pSTAT3 marked a subgroup of reactive astrocytes that appeared to promote brain metastasis in both mouse models and human clinical samples [26]. These pSTAT3^+^ reactive astrocytes blocked the access of CD8^+^ cytotoxic T cells to cancer cells (Fig. 1a(a)) by the upregulating of immunosuppressive molecules such as programmed cell death–1 ligand 1 (PD-L1), vascular endothelial growth factor–A (VEGF-A), lipocalin-2, and tissue inhibitor of metalloproteinases–1 (TIMP-1) [26]. Tumor-associated astrocytes in primary brain tumors were also recently found to express STAT3 and PD-L1 at high levels and to confer an immunosuppressive environment through increased production of cytokines such as interleukin (IL)–10 and transforming growth factor– β (TGF-β) [28]. CD74-positive microglia, the resident macrophage-like cells of the central nervous system (CNS), promote the growth of primary brain tumors through suppression of the antitumor immune response [29], and STAT3^+^ reactive astrocytes associated with brain metastases showed increased expression of the CD74 ligand MIF (macrophage migration inhibitory factor) and increased binding to CD74^+^ microglia (Fig. 1a(b)). The microglia were thus activated by STAT3^+^ reactive astrocytes via the MIF-CD74 axis and showed upregulation of midkine, a downstream target of the nuclear factor (NF)–κB signaling pathway that promotes the development of brain metastases [26]. (Fig. 1a(c)). Cross talk between microglia and reactive astrocytes thus contributes to the establishment of an immunosuppressive microenvironment (Fig. 1a(d)) and thereby supports brain metastasis of breast cancer, with the STAT3 signaling pathway being a potential therapeutic target for intervention in this process.

Reactive astrocytes also protect cancer cells from chemotherapy by upregulating the expression of survival genes in the cancer cells in a manner dependent on gap junctions between the two cell types [30, 31]. The formation of such gap junctions is mediated by the interaction of protocadherin 7 (PCDH7) on cancer cells with connexin43 (Cx43) on astrocytes (Fig. 1a(e)). Cancer cells activate the cGAS–STING (stimulator of interferon genes) pathway in astrocytes by transfer of 2′,3′-cyclic GMP-AMP (cGAMP) through gap junctions and thereby promote the production of inflammatory cytokines such as interferon-α (IFN-α) and tumor necrosis factor–α (TNF-α) by the astrocytes (Fig. 1a(f)). These cytokines then activate STAT1 and NF-κB signaling pathways in cancer cells and thereby support brain metastasis [32] (Fig. 1a(g)).

### PI3K-Akt signaling pathway

The phosphoinositide 3-kinase (PI3K)–Akt pathway is a key intracellular signaling pathway that promotes various cellular processes including proliferation, survival, metabolism, and angiogenesis in response to extracellular signals that activate receptor tyrosine kinases or G protein–coupled receptors. The PI3K-Akt signaling pathway has also been implicated as a major regulator of brain metastasis (Fig. 1b). The activation of PI3K was detected in a large proportion (77%) of brain metastases in breast cancer patients [33], and activation of PI3K-Akt signaling in such metastases has been associated with a poor survival outcome [34, 35]. The PI3K-Akt signaling pathway contributes to upregulation of the expression of immunosuppressive or metastasis-promoting genes such as those for PD-L1, cytotoxic T lymphocyte–associated protein 4 (CTLA4), colony-stimulating factor 1 (CSF1), and the CSF1 receptor (CSF1R) in cancer cells or microglia in the microenvironment of brain metastases [33] (Fig. 1b(h, i)). Pharmacological inhibition of PI3K activity was found to attenuate the expression of these genes as well as the infiltration of metastatic breast cancer cells in the brain of mice [33].

PI3K class I enzymes include four catalytic subunit isoforms, with the α and β isoforms often being overexpressed in breast cancer cells [36] and the γ and δ isoforms being preferentially expressed in immune cell types, including macrophages and microglia [37]. PI3Kγ signaling in tumor-associated macrophages (TAMs) has been shown to support tumor growth by promoting immune suppression in head and neck squamous cell carcinoma [38] (Fig. 1b(j)). CSF1 signaling in TAMs promotes both the invasiveness and intravasation of breast cancer cells [39], and inhibition of such signaling-attenuated tumor growth by reducing the number of TAMs and increasing the infiltration by CD8^+^ T cells in mouse models of breast and cervical cancer [40].

Loss of PTEN (phosphatase and tensin homolog), a negative regulator of PI3K-Akt signaling, has been detected in 25% to 71% of brain metastases in breast cancer patients and occurs preferentially in TNBC [34, 35, 41]. Loss of PTEN expression was detected specifically in metastatic breast cancer cells in the brain, not in those in other organs, as a result of epigenetic regulation by miRNAs derived from astrocytes [41] (Fig. 1b(k)). Overexpression of PTEN was shown to attenuate the invasiveness and migration of breast cancer cells as well as astrocyte activation [34]. PTEN loss-activated NF-κB signaling and increased expression of the chemokine CCL2 in breast cancer cells (Fig. 1b(l), and the CCL2^+^ cancer cells recruited macrophages expressing the receptor for CCL2 (CCR2) (Fig. 1b(m)), resulting in the promotion of brain metastasis outgrowth after cancer cell extravasation [41].

Mammalian target of rapamycin (mTOR) is a downstream effector of the PI3K-Akt pathway, and its activity in breast cancer has been shown to mediate resistance to PI3K inhibition. Combined inhibition of PI3K and mTOR was able to overcome resistance to a PI3K inhibitor in an orthotopic model of brain metastasis by HER2-positive breast cancer [42].

### HER2-HER3 signaling

HER2-positive breast cancer shows a susceptibility to brain metastasis similar to that of TNBC. Formation of HER2-HER3 heterodimers results in marked activation of PI3K-Akt signaling in breast cancer cells [43]. The expression of HER3 was found to be increased in a *HER2*-amplified breast cancer cell line (BT-474) after implantation of the cells into the mouse brain [44]*.* Immunohistochemistry revealed that HER3 was overexpressed in 60% of brain metastases in breast cancer patients [45], whereas tissue microarray analysis showed that 57.6% of brain metastases in patients with various types of solid tumor—including HER2^+^ and HER2^−^ breast cancer, lung cancer, and colon cancer—were positive for phosphorylated (activated) HER3 [46]. Resistance to PI3K inhibition in brain metastases of breast cancer was rescued by inhibition of HER3 activity both in vitro and in vivo [47], suggesting that activation of the PI3K-Akt pathway by HER3 contributes to brain metastasis. A HER3 inhibitor U3–1402, which is a HER3-targeted antibody drug conjugate, is currently under investigation in patients with metastatic breast cancer positive for HER3 overexpression (NCT02980341).

## Potential strategies for prevention or treatment of brain metastasis in breast cancer

### Molecularly targeted therapy

Although HER2-targeted agents including trastuzumab, pertuzumab, and trastuzumab plus emtansine (T-DM1) have failed to prevent brain metastasis in breast cancer patients [48–50], various regimens have shown promise for the treatment of established brain metastases. Combination therapy with novel anti-HER2 agents plus capecitabine has thus shown efficacy in HER2^+^ breast cancer patients with brain metastases. Neratinib is a pan-HER tyrosine kinase inhibitor (TKI) that binds irreversibly to HER1, HER2, and HER4, and the combination of neratinib plus capecitabine showed a CNS response rate of 49% in such patients, compared with a value of only 8% for neratinib monotherapy [51]. Tucatinib is another TKI that is highly specific for HER2. The effects of tucatinib in patients with HER2–positive metastatic breast cancer who have disease progression after therapy with multiple HER2-targeted agents have been reported on [52]. In that report, addition of tucatinib to the combination of trastuzumab and capecitabine showed increased CNS response rates and better progression-free survival (PFS) rates in patients with HER2^+^ breast cancer and brain metastases.

An inhibitor of cyclin-dependent kinases (CDKs) 4 and 6 has also shown promise for the treatment of brain metastases in breast cancer patients. The combination of this inhibitor, abemaciclib, with endocrine therapy was thus found to be effective in patients with hormone receptor–positive, HER2-negative breast cancer and brain metastases, with 38% of patients showing a decrease in the metastatic tumor load [53].

Given that cross talk between metastatic cancer cells and their microenvironment is implicated in the development of brain metastases of breast cancer, therapy targeted to the microenvironment or to such cross talk is also under investigation. The STAT3 inhibitor silibinin, which crosses the blood-brain barrier [54], has thus been shown to impair the viability of brain metastases in both mice and humans [26]. This inhibitor is thought to block the growth of brain metastases by targeting STAT3 in tumor-associated astrocytes and thereby attenuating their cross talk with cancer cells and microglia. The JAK inhibitor ruxolitinib limited the growth of primary brain tumors as well as reduced the number of activated tumor-associated astrocytes marked by STAT3 phosphorylation in mice [28]. Gap junctions between cancer cells and reactive astrocytes are another potential therapeutic target for brain metastases, with orally bioavailable modulators of gap junctions (meclofenamate and tonabersat) having been found to inhibit brain metastatic outgrowth [32].

### Immune checkpoint therapy

Immune checkpoint inhibitors have been approved for the treatment of lung cancer and melanoma. The immune checkpoint protein PD-L1 is expressed on the surface of cancer cells and induces exhaustion or apoptosis in tumor-infiltrating cytotoxic T cells through interaction with programmed cell death–1 (PD-1) expressed on their surface [55]. Pembrolizumab was the first PD-1 inhibitor shown to be effective against previously untreated brain metastases in patients with melanoma or non–small cell lung cancer, with CNS response rates of 22% and 33%, respectively [56]. Another monoclonal antibody to PD-1, nivolumab, has shown an efficacy similar to that of pembrolizumab for previously untreated brain metastases in patients with melanoma, with a CNS response rate of 20% [57]. The combination of nivolumab and ipilimumab, a monoclonal antibody to the immune checkpoint protein CTLA4, has shown the most impressive CNS response rate (52%) to date for untreated brain metastases in melanoma patients, with the value being 26% for intracranial complete responses [58]. Recent advances in immune checkpoint therapy have thus provided additional potential therapeutic options for patients with TNBC and brain metastases, with pembrolizumab and the anti–PD-L1 antibody atezolizumab now being available for some such patients.

The mechanisms underlying the effectiveness of immunotherapy for brain metastases are under investigation. The number of FOXP3^+^ regulatory T cells has been found to increase in association with the progression of brain metastases [59]. Immune checkpoint inhibitors that target PD-1 or PD-L1 are thought to reactivate the effector function of cytotoxic T cells rendered exhausted by the PD-1–PD-L1 signaling axis [60].

### Novel therapeutic targets

To identify novel and specific mechanisms of brain metastasis and thereby provide insight into prevention or treatment of this condition, we developed mouse xenograft models of brain metastasis based on intracardiac injection of human breast cancer or melanoma cell lines and performed RNA-sequencing analysis of both brain metastases and matched primary tumors [61]. The sequence data were mapped to the corresponding human and mouse genomic DNA sequences in order to identify genes in mouse brain tissue (the tumor microenvironment), and the human cancer cells whose expression was associated specifically with metastasis. We found that expression of the mouse genes *Tph2*, *Sspo*, *Ptprq*, and *Pole* was specifically upregulated in brain tissue harboring metastases, whereas that of the human genes *CXCR4*, *PLLP*, *TNFSF4*, *VCAM1*, *SLC8A2*, and *SLC7A11* was specifically upregulated in brain-metastasizing cancer cells. Further characterization of such novel metastasis-associated genes and their interactions may eventually lead to advances in therapy that improve the prognosis of cancer patients. Investigation of the influences of immune cells on brain metastasis will require the development of improved experimental models with an intact immune system.

Among the human genes whose expression was specifically upregulated in brain metastatic tumor cells of our mouse xenograft models, *SLC7A11* is of particular interest. The *SLC7A11* gene encodes the xCT subunit of system xc(−), a sodium-independent cystine-glutamate antiporter that mediates the uptake of cystine into cells in exchange for intracellular glutamate [62]. Expression of xCT at the cell surface is essential for the uptake of cystine required for intracellular glutathione synthesis and is thus an important determinant of intracellular redox balance [63]. Expression of xCT thus protects cells from reactive oxygen species (ROS) and plays a role in suppression of an iron-dependent form of ROS-induced cell death known as ferroptosis. Studies of cancer stemlike cells that express a variant isoform of CD44 (CD44v) have revealed that CD44v interacts with xCT and thereby stabilizes its localization at the cell surface and confers resistance to therapy-induced oxidative stress [64, 65]. Furthermore, the CD44v-xCT axis in metastatic breast cancer cells was found to confer protection against ROS and thereby to promote lung metastasis in mice [66]. Together, these findings suggest that xCT is a key molecule for the ability of metastatic cells to colonize and grow in the brain and lung, and it is therefore a potential therapeutic target.

Sulfasalazine, a drug that has been administered for the treatment of inflammatory bowel disease and rheumatoid arthritis [67], has also been found to inhibit xCT-dependent cystine transport [68] and to induce ferroptosis in cancer cells [65]. Physician-initiated clinical trials of sulfasalazine either alone or in combination with anticancer drugs have been performed for gastric [69] and lung [70] cancer, respectively. Single-agent treatment was associated with a reduction in the number of CD44v^+^ cancer stemlike cells, and the combination treatment with a significant increase in progression-free survival. Whether the promise of such xCT-targeted therapy may extend to the prevention or treatment of breast cancer metastasis warrants further investigation.

## Future prospects

In addition to the treatment of patients with brain metastases, prevention of brain metastasis from the primary tumor is an important clinical goal. Such prevention will require the detection of circulating brain-tropic cancer cells before their extravasation. Liquid biopsy is a potential screening tool for the detection of such cells in the circulation [71]. The gene signature of circulating tumor cells (CTCs) associated with brain metastasis of breast cancer has revealed the up-regulation of Notch signaling and NF-κB signaling [71]. Newly developed techniques for the detection of CTCs and cell-free tumor DNA in cerebrospinal fluid can also potentially be applied to the detection of cancer cells that can grow in the brain [72].

Traditional cytotoxic agents and anti-HER2 agents have a limited role in the management of brain metastases in breast cancer patients. Intrathecal injection of methotrexate is adopted in the clinic as a therapeutic option for leptomeningeal metastasis in breast cancer. A case report showed that intrathecal trastuzumab was also a safe and effective therapy for HER2-positive breast cancer with leptomeningeal metastasis [73]. Such intrathecal treatment warrants investigation for its ability to prevent the development of brain macrometastases in patients positive for CTCs or cell-free tumor DNA in cerebrospinal fluid or for a brain-tropic CTC gene signature in blood.

We previously found that the heterogeneity of HER2 expression among breast cancer cells is associated with poor survival in mice with brain metastases [74]. Furthermore, conversion of the expression of HER2 as well as hormone receptors has been observed in metastasized breast cancer. For instance, conversion of HER2, from positive to negative, occurred in 14% of patients by comparing primary tumors with brain metastasis of breast cancer [75]. These observations suggest the potential risk of conversion and/or heterogeneity of HER2 expression during the course of anti-HER2 treatments.

TNBC has the worst prognosis among breast cancer subtypes as a result of its rapid progression and lack of a conventional therapeutic target. TNBC with symptomatic brain metastasis is preferentially treated with radiation therapy rather than systemic therapy. Patients with brain metastases manifest various symptoms including headache (61.9%), nausea and vomiting (45.7%), visual disorders (26.3%), seizures (30.4%), and motor dysfunction (46.6%) [76]. Corticosteroids are usually administered to reduce these neurological symptoms during radiation therapy as well as after treatment. Patients with TNBC are at a disadvantage compared with those with other subtypes of breast cancer in that they need to wait to start systemic chemotherapy until corticosteroid treatment has been discontinued because of the risk of infection. In contrast, patients with HER2^+^ or luminal breast cancer have other therapeutic options, given that anti-HER2 agents and endocrine therapy can be administered concurrently with corticosteroids and radiation therapy. Immune checkpoint inhibitors are a promising therapeutic option for TNBC patients with brain metastases before radiation therapy, although further studies are required to support this option.
